# Multimodal Imaging-Based Potential Visualization of the Tumor Microenvironment in Bone Metastasis

**DOI:** 10.3390/cells10112877

**Published:** 2021-10-25

**Authors:** Jang Bae Moon, Su Woong Yoo, Changho Lee, Dong-Yeon Kim, Ayoung Pyo, Seong Young Kwon

**Affiliations:** 1 Department of Nuclear Medicine, Chonnam National University Medical School and Hwasun Hospital, Hwasun-gun 58128, Korea; h-dfj@hanmail.net (J.B.M.); yoosw.md@gmail.com (S.W.Y.); ch31037@chonnam.ac.kr (C.L.); 2 College of Pharmacy and Research Institute of Pharmaceutical Science, Gyeongsang National University, Jinju 52828, Korea; blueburr@gmail.com; 3 Accelerator & RI Development Team, Korea Atomic Energy Research Institute, Daejeon 56212, Korea; aypyo@kaeri.re.kr

**Keywords:** bone metastasis, microenvironment, imaging, metabolism

## Abstract

Bone metastasis (BM) is the most common malignant bone tumor and a significant cause of morbidity and mortality for patients with cancer. Compared to other metastatic organs, bone has unique characteristics in terms of the tumor microenvironment (TME). Precise assessments of the TME in BM could be an important step for developing an optimized management plan for patient care. Imaging approaches for BM have several advantages, such as biopsy not being required, multiple site evaluation, and serial assessment in the same sites. Owing to the developments of new imaging tracers or imaging modalities, bone TME could be visualized using multimodal imaging techniques. In this review, we describe the BM pathophysiology, diagnostic principles of major imaging modalities, and clinically available imaging modalities to visualize the TME in BM. We also discuss how the interactions between various factors affecting the TME could be visualized using multimodal imaging techniques.

## 1. Introduction

Bone is one of the major target sites for metastasis. Bone metastasis (BM) is the most common malignant bone tumor and a significant cause of morbidity and mortality for patients with cancer. BM often causes critical skeletal-related events (SREs), including pain, fractures, hypercalcemia, and spinal cord compression, which contribute to reduced quality of life and decreased survival [1,2,3].

For the management of BM, variable therapeutic or management tools, such as surgery, radiotherapy, systemic therapy, and bone-targeted agents (bisphosphonate and denosumab), are available worldwide to minimize SREs and improve quality of life [4,5,6,7,8]. However, their reliability and effectiveness remain unclear. This could be owing to the lack of understanding of the tumor microenvironment (TME) comprising tumor cells, stromal cells, and bone matrix [9,10]. Compared with other metastatic organs, bone has unique characteristics for the TME [11,12]. Precise assessments of the TME in BM could be important to develop an optimized management process for patient care.

Medical imaging has been used for identifying metastatic sites, guiding biopsy sites, and evaluating the therapeutic responses, thus permitting further management plans to be drawn for patients with BM [13]. Owing to the developments of new imaging tracers or imaging modalities, several studies have been performed for visualization of the TME [14,15,16,17]. Compared to conventional methods to assess the TME, imaging approaches have several advantages, such as biopsy not being required, evaluation of multiple sites, and serial assessment at the same sites.

In this review, we describe the BM pathophysiology and diagnostic principles of major imaging modalities. Then, we classify factors related to the TME in BM into two categories (primary tumor and bone tissues) and introduce multimodal imaging patterns according to each category.

## 2. Imaging-Based Understanding of BM Pathophysiology

### 2.1. Pathophysiologic Factors Related to Bone Metastasis

The process of BM includes multiple steps, namely neoangiogenesis, intravasation, and extravasation [18]. Angiogenesis is necessary to provide nutritional support to the tumor cells, thus enabling their migration. Before migration, tumor cells may undergo epithelial-to-mesenchymal transition (EMT), which is a reversible switch from an epithelial to a mesenchymal cellular phenotype [19]. EMT occurs in variable pathological conditions, such as chronic inflammation, wound healing, tumor progression, and metastases. This functional modification, which includes the change from a polarized tight junction type to a flat, spindled, and motile type, enables the tumor cells to drive the invasion of the basement membrane (intravasation), enter the blood vessels, and migrate to distant sites [19]. On reaching the bone tissue, tumor cells adhere to the vessel endothelium, extravasate, and bind to bone stromal cells and bone matrix through specific cell adhesion molecules [20]. Bone is a large repository for growth factors (insulin-like growth factor, platelet-derived growth factor, and fibroblast growth factor), which are released during bone resorption and promote bone homing, colonization, and subsequent tumor cell proliferation [21]. Additionally, tumor cells may proliferate as an effect of mesenchymal-to-epithelial transition [22].

Bone maintains a dynamic balance between osteoblastic (bone-forming) and osteoclastic (resorptive) activity, which undergoes bone modifications within the physiological process of bone remodeling in normal conditions [13,23,24]. This complex process is regulated by resident bone cells and other cell types, including lymphocytes, macrophages, hematopoietic cells, and endocrine signaling molecules, in bone tissues [25]. When tumor cells proliferate in bone tissues, osteoblastic and osteoclastic changes occur simultaneously [26]. The simultaneous presence of both cell types elucidates the reason why BMs could show osteolytic, osteosclerotic (osteoblastic), or mixed lesions [27]. Osteolytic metastases involve osteoclast-mediated bone resorption and are frequently observed in thyroid, lung, and renal carcinomas [13]. In osteolytic metastases, the interaction between the receptor activator of nuclear factor kappa B (RANK) and its ligand (RANKL) plays a consistent role [28]. RANKL levels lead to hyperactivation of osteoclastogenesis and bone resorption, permitting metastatic clones to invade the bone tissues. Liver specimens of hepatocellular carcinoma (HCC) patients with BM express high RANKL levels, suggesting a potential role of the RANK/RANKL axis in the formation of osteolytic BM [29]. Activated osteoclasts reabsorb the bone by producing hydrochloric acid and metalloproteases, which dissolve the mineral in bone and cause breakdown of the collagenous matrix [30]. Osteoblastic metastases involve the formation of pathologic new bone, particularly in prostate cancer [13]. The osteoblastic component of a lytic metastasis represents the reaction of normal bone to the metastatic process [23]. Breast cancer is one of the solid tumors with mixed patterns in BM.

### 2.2. Diagnostic Principle for Imaging Modalities

**Figure 1:**
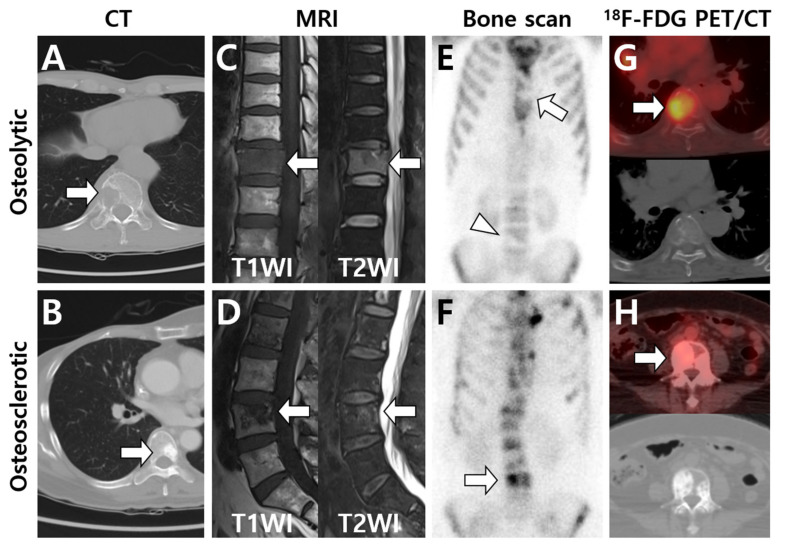
Typical imaging patterns of bone metastases observed on major imaging modalities. According to computed tomography (CT) findings, osteolytic or osteosclerotic metastases are initially placed in the upper and lower rows, and imaging findings are shown for each device. (**A**) Osteolytic metastasis destructs cortex and cancellous bone structures and is observed as a radiolucent lesion on CT (arrow). (**B**) Osteosclerotic metastasis forms new bone in the marrow space and is observed as a radiopaque lesion on CT (arrow). (**C**,**D**) On T1-weighted MRI, the signal intensity (SI) is low in both osteolytic (**C**) and osteosclerotic (**D**) metastases (arrows) as the normal bone marrow is replaced with tumor cells. In contrast, T2-weighted MRI shows high SI in osteolytic metastasis (**C**) and heterogeneous SI in osteosclerotic metastasis (**D**) (arrows). (**E**) Osteolytic metastasis appears as decreased radiotracer uptake on bone scan (arrow and arrowhead). (**F**) Osteosclerotic metastasis shows increased radiotracer uptake on bone scan (arrow). (**G**) ^18^F-FDG PET/CT generally shows increased FDG uptake in osteolytic metastasis (arrow), although FDG uptake could also be affected by histologic subtypes of the primary tumor. (**H**) In contrast, osteosclerotic metastasis is depicted as a relatively low FDG-avid lesion (arrow) on ^18^F-FDG PET/CT. (**A**,**C**,**E**: hepatocellular carcinoma; **B**,**D**,**F**,**H**: intraductal breast carcinoma; **G**: lung adenocarcinoma).

In clinical aspects, BM imaging methods can be generally divided into two categories based on detection mechanisms, namely bone-marrow- or tumor-specific methods and osteoblastic-activity-related methods [31]. Bone-marrow- or tumor-specific methods include ^18^F-fluorodeoxyglucose (^18^F-FDG) positron emission tomography (PET) and magnetic resonance imaging (MRI). Imaging modalities that reflect osteoblastic activity include radiography, computed tomography (CT), and bone scintigraphy (or bone scan) [32]. The combination of nuclear imaging modalities using either bone- or tumor-specific radiotracers with CT or MRI has been actively used in the clinic, namely single-photon emission computed tomography (SPECT)/CT, PET/CT, or PET/MRI [31,32]. Typical imaging patterns among four major modalities are illustrated in Figure 1.

CT can show the detailed characteristics of bone tissue and detect metastases within the bone marrow before bone destruction occurs [27]. In particular, CT is useful in visualizing the bone cortical integrity, permitting an easy characterization of the lesions as osteolytic or osteosclerotic [20]. The lesion on CT can be visualized as an increased attenuation of the normal fatty bone marrow. The lesion is abnormal when an attenuation difference between the right and left extremities is more than 20 Hounsfield units (HU) [33]. Nevertheless, the CT appearance of the lesion could be variable, depending on the balance between bone-forming and resorptive processes. Dual-energy CT can improve the diagnostic performance for the detection of BM through better characterization of different matter components, especially for medullary bone lesions [34,35]. CT also has advantages compared with radiography and bone scintigraphy in that it may be used to identify lesions in the spine and calvarium and to perform an image-guided biopsy. Through the clinical application of hybrid scanners, such as PET/CT or SPECT/CT, CT information about morphologic characteristics has contributed to improving the differential diagnosis and response evaluation after therapy [31].

Bone scintigraphy (or bone scan) with ^99m^Tc-labeled diphosphonate is the most widely used method of detecting BM. ^99m^Tc-methylene diphosphonate (^99m^Tc-MDP) is the most commonly used tracer. This radiotracer shows rapid clearance from blood and soft tissues, which results in good image contrast as early as 2 h after injection [36]. It accumulates in areas of increased osteoblastic activity and increased blood flow and has an advantage for detecting osteoblastic metastases, particularly in breast or prostate cancers [23]. In contrast, bone scintigraphy is less sensitive for detecting BM that has little osteoblastic reaction or aggressive lesions with osteolysis (osteolytic metastases), which can show decreased radiotracer uptake [23]. Osteolytic metastases with little osteoblastic reaction are frequently overlooked in specific types of cancers, such as thyroid cancer or HCC. Tracer uptake of the BM lesion could also be variable according to the balance between bone-forming and resorptive processes, particularly in breast cancer. However, the diagnostic performance can be limited as radiotracer uptake can occur in any area of high bone turnover related to trauma, infection, or inflammation [37]. To evaluate false-positive findings, correlative imaging modalities with CT, MRI, or PET/CT should be considered [13]. While bone scan appears as planar images, SPECT acquires images in a cross-sectional method after administration of the same radiotracer, which can provide improved anatomic localization and diagnostic accuracy [27]. SPECT/CT can be useful for the differential diagnosis of indeterminate findings on a bone scan [13,27]. 

MRI shows high sensitivity for BM detection as it can provide detailed information of marrow abnormalities [38]. Normal bone marrow contains a high percentage of fat tissues, which can be shown as high signal intensity (SI) on T1-weighted images (T1WIs) [13]. In BM, normal marrow fat cells are replaced with tumor cells, showing low SI on T1WIs. On T2-weighted images (T2WIs), the SI is generally higher in BM than in the normal bone marrow owing to high water content [37,39]. MRI is also highly helpful for the assessment of epidural, nerve, and spinal cord involvement through superior soft-tissue contrast [40,41], permitting differential diagnosis between benign and malignant vertebral compression fractures and evaluation of spinal cord compression [20]. In contrast, MRI has a drawback for the evaluation of cortical bone destruction as the cortical bone is demonstrated as a dark signal on both T1- and T2-weighted images [27,37]. MRI with diffusion-weighted imaging (DWI) is a promising imaging technique in the evaluation of BM from other solid tumors [42]. Through the combination of functional information from DWI with morphological MR sequences, differences between the cellularity of BM and normal marrow can be evaluated. MRI with dynamic contrast enhancement (DCE) has recently been investigated for the diagnosis of spinal BM, showing the ability to detect vertebral body infiltration and tumor vascularity [43]. The latter characteristic could permit MRI with DCE to contribute to evaluation of the response to antiangiogenic therapies [20]. DWI and DCE parameters of BM changed significantly in patients with HCC after radiation therapy (RT) [44]. In addition, the percent change in apparent diffusion coefficient (ADC) was related to therapeutic responses and local tumor progression after RT in BM.

^18^F-FDG PET/CT showed good diagnostic performance for the detection of BM [38]. ^18^F-FDG is a glucose analog that is taken up by tumor cells and phosphorylated and becomes trapped within the tumor cells. Tumor or inflammatory cells, which have a high glucose consumption, show high FDG uptake through increased expression of glucose transporter 1 (Glut1) and hexokinase concentration [13]. ^18^F-FDG PET/CT can detect the primary or metastatic tumor tissues with high glucose uptake [45]. Although ^18^F-FDG PET/CT has been reported to detect osteolytic, osteoblastic, and mixed lesions, it showed high sensitivity for detecting lytic metastases compared with bone-seeking radiotracers, such as ^99m^Tc-labeled diphosphonate and ^18^F-sodium fluoride [13,31]. ^18^F-FDG PET/CT is also more sensitive in evaluating bone marrow involvement and early BM lesions. In particular, ^18^F-FDG PET/CT is useful to identify the primary tumor and biopsy sites in patients with suspicious BM from unknown primary sites [46]. In contrast, its specificity can be low for the differential diagnosis between BM and active inflammatory lesions, such as infectious spondylitis or recent traumatic fracture, as ^18^F-FDG can be taken up by inflammatory as well as tumor cells.

## 3. Imaging Patterns Based on Primary Tumor Characteristics

Although the clinically available imaging modalities described above have limitations in precisely understanding the TME, clinical values must be derived by combining the conventional imaging modalities with new molecular imaging technologies. In the next two sections, we introduce multimodal imaging techniques to visualize the TME in BM according to two factors, namely primary lesion and bone microenvironment.

### 3.1. Multimodal Imaging According to the Primary Tumor Site and Histologic Type

Because the BM characteristics can be affected by the primary tumor, the BM lesion may have various image patterns depending on the primary cancer [31]. Major imaging findings in BM are summarized in Table 1. When the primary lesion is located in the thyroid, lung, liver, or kidney, osteoclast activation and its interaction with the bone stroma lead to an osteolytic pattern on CT [47,48,49,50] and low SI in T1WI on MRI [51,52,53,54]. ^18^F-FDG PET/CT generally depicts high FDG uptake in BM lesions from the lung [45,55,56], whereas those from the liver or kidney show low FDG uptake [57,58], which is related to FDG avidity of the primary lesion to some extent. In BM from breast, osteoblastic activity dominates during the early stages of BM before the initiation of the osteolytic phase, and a mixed pattern is shown on CT according to the balance work between osteoblast and osteoclast activation [59]. On MRI, BM from breast shows low SI in T1WI and various SI in T2WI [38]. In bone scan, radiotracer uptake is generally increased by osteoblastic activity and secondary bone changes [60,61]. There is generally high FDG uptake in BM lesions from the breast due to cancer and bone stromal cells interacting with the cancer cells [62,63]. BM lesions from prostate cancer generally demonstrate osteosclerotic patterns with high HU on CT due to the osteoblastic-dominated response [64] and are more sensitive on the bone scan than on ^18^F-FDG PET/CT [65,66].

Even if the primary tumor originates from the same organ, the metabolic phenotype of the primary tumor can be different depending on the histologic subtype, which may affect the imaging pattern of BM lesions on ^18^F-FDG PET/CT. Among the histologic subtypes of lung cancer, FDG uptake in tumor cells is generally higher in lung squamous cell carcinoma than in lung adenocarcinoma as Glut1 is highly expressed mainly in the squamous cell membrane, whereas Glut1 expression in lung adenocarcinoma is distributed in the cytosol [85]. Clinically, the diagnostic performance of ^18^F-FDG PET/CT for BM detection could be limited in lung adenocarcinoma with low FDG avidity. Among the histologic subtypes of breast cancer, there is a difference in FDG uptake between invasive ductal and invasive lobular carcinomas, and this difference could affect the metabolic phenotype in BM lesions and sensitivity in PET/CT between the subtypes [86].

### 3.2. Multimodal Imaging According to the Differentiation Status of the Primary Tumor

Even if the primary tumor has the same histologic type, the imaging patterns in BM could vary depending on the differentiation status or metabolic phenotype of the primary tumor. Multimodal imaging modalities using different radiotracers can potentially visualize the TME in BM, reflecting the differentiation status.

**Figure 2:**
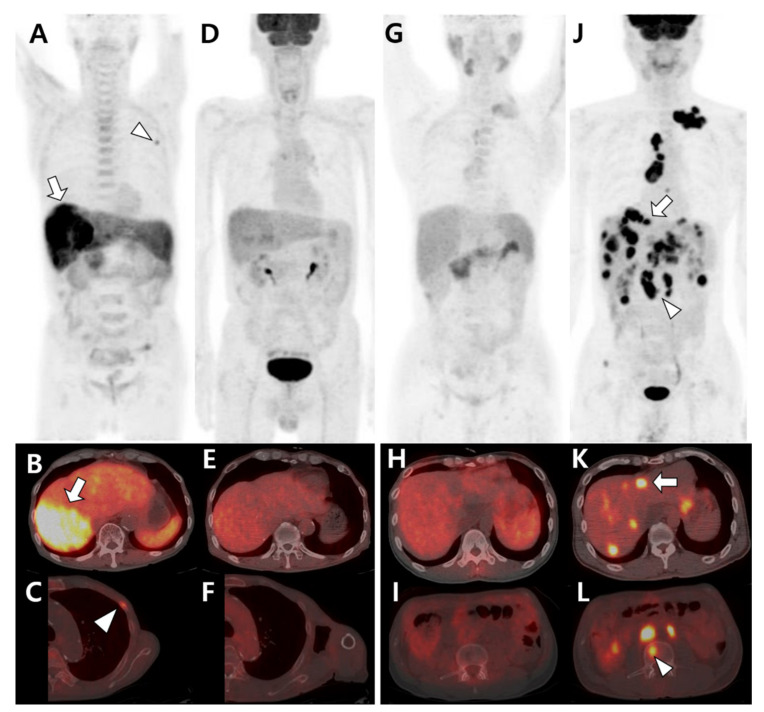
Different tracer avidities of primary and metastatic sites in patients with hepatocellular carcinoma (HCC). (**A**–**F**) A 77-year-old male patient with well-differentiated HCC. ^11^C-acetate positron emission tomography (PET)/computed tomography (CT) (**A**–**C**) shows high acetate uptake in a primary hepatic tumor (arrow in **A**,**B**) and metastatic bone lesion in the left third rib (arrowhead in **A**,**C**). However, ^18^F-fluorodeoxyglucose (^18^F-FDG) PET/CT (**D**–**F**) shows no significant uptake in the related sites. (**G**–**L**) A 43-year-old male patient with poorly differentiated HCC. ^11^C-acetate PET/CT (**G**–**I**) shows mild uptake or isometabolism in the hepatic tumor (**H**) and metastatic bone lesion (**I**). In contrast, ^18^F-FDG PET/CT (**J**–**L**) shows intense FDG uptake in hepatic tumors (arrow in **J**,**K**) and multiple metastatic lesions in the lymph nodes and bones (arrowhead in **J**,**L**).

HCC is one of the highly heterogeneous cancers, which has several implications in evaluating the TME of metastatic lesions. Even in the same tumor mass of a single patient, the imaging pattern can be heterogeneous depending on the degree of tumor differentiation. There is a relationship between the degree of tumor differentiation and DCE patterns on MRI [87,88,89]. In well-differentiated HCC, MRI shows a relatively hypoenhanced pattern on arterial phase and high SI on hepatobiliary phase. In contrast, in HCC with poor differentiation, a hyperenhancement pattern on arterial phase and low SI on hepatobiliary phase are observed. When the tumor mass in HCC comprises tumor cells with various degrees of differentiation, the enhancement patterns also varies in each phase [88,90]. Many studies have been reported using ^11^C-acetate PET/CT, which reflects lipid metabolism, as well as ^18^F-FDG PET/CT in diagnosing primary HCC and metastatic tumors [91,92]. These two radiotracers are mutually complementary owing to different radiotracer avidities according to tumor differentiation, with high ^11^C-acetate and FDG uptake found in well-differentiated and poorly differentiated tumors, respectively [92]. Moreover, metastatic tumors could also reflect the characteristics of the primary tumor to some extent; therefore, the tracer avidity of metastatic tumors, including BM, also changes depending on that of the primary tumor (Figure 2).

**Figure 3:**
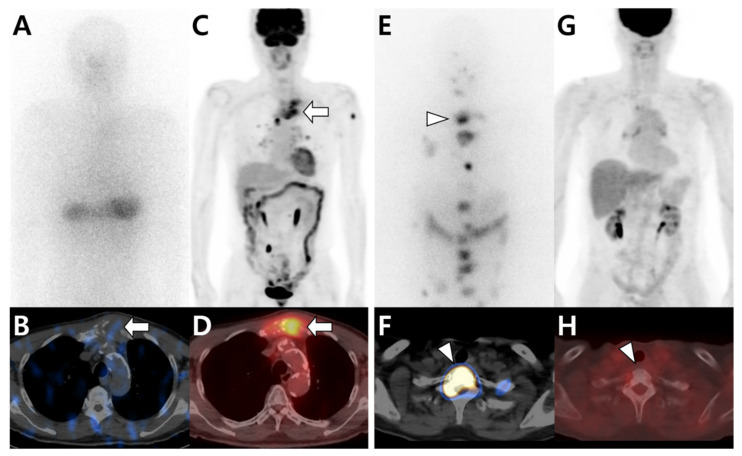
Different tracer avidities in bone metastases from papillary thyroid carcinoma (PTC) according to differentiation status. (**A**–**D**) A 79-year-old male patient with PTC. ^131^I whole-body scan (**A**) and single-photon emission computed tomography (SPECT)/computed tomography (CT) (**B**) show little iodine uptake in metastatic lesions located in the sternum (arrow). In contrast, ^18^F-fluorodeoxyglucose (^18^F-FDG) positron emission tomography (PET)/CT (**C**,**D**) shows intense FDG uptake in the sternum (arrow). (**E**–**H**) A 66-year-old female patient with PTC. ^131^I whole-body scan (**E**) and SPECT/CT (**F**) show multiple iodine uptake in metastatic bone lesions, including those in the T1 vertebra (arrowhead). However, ^18^F-FDG PET/CT (**G**,**H**) shows no significant uptake at the same site.

Although differentiated thyroid cancer (DTC), such as papillary or follicular cancer, has an excellent prognosis, the prognosis deteriorates rapidly when distant metastasis occurs [20]. Bone is the second most common site of distant metastasis in DTC [70]. Sodium–iodide symporter (NIS) expressed on the cell membrane is an important tool for the diagnosis (^123^I) and therapy (^131^I) of DTC. NIS expression in tumor cells is maintained in the early phase of distant metastasis, where ^131^I therapy contributes to patient outcomes [93]. However, when the degree of differentiation is poor, the expression level of NIS and iodine uptake decreases. Conversely, glucose metabolism, which is visualized as FDG uptake on ^18^F-FDG PET/CT, increases (Figure 3) [94]. This flip-flop phenomenon is important for further management plans for patients with distant metastasis, including BM, as ^131^I therapy is highly limited in DTC patients with poor differentiation.

## 4. Imaging Patterns Based on the Bone Microenvironment

When tumor cells metastasize to bone, they affect the TME in various ways through interaction among tumor cells, stromal cells, and bone matrix [95]. Molecular imaging is applied to visualize the TME using radiotracers for specific targets, such as tumor metabolism, receptor expression, and stromal cell activation, in BM (Table 2). Additionally, multimodal imaging techniques using these radiotracers can provide an opportunity to noninvasively assess how tumor cells interact with various stromal cells or bone matrices affecting the TME.

**Figure 4:**
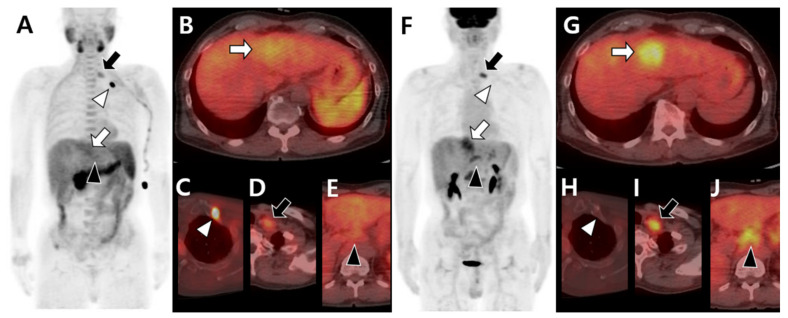
Different tracer avidity according to the metastatic organ in the same patient with hepatocellular carcinoma before therapy. ^11^C-acetate positron emission tomography (PET)/computed tomography (CT) (**A**–**E**) shows mild uptake in the primary hepatic tumor (white arrow in **A**,**B**). The metastatic bone lesion in the left first rib shows intense acetate uptake (white arrowhead in **A**,**C**) but less acetate uptake in metastatic lymph nodes of the left supraclavicular (black arrow in **A**,**D**) and common hepatic (black arrowhead in **A**,**E**) areas. ^18^F-fluorodeoxyglucose (^18^F-FDG) PET/CT (**F**–**J**) shows intense uptake in primary hepatic tumors (white arrow in **F**,**G**). There is no significant FDG uptake in the left first rib (white arrowhead in **F**,**H**) but intense uptake in the left supraclavicular (black arrow in **F**,**I**) and common hepatic (black arrowhead in **F**,**J**) lymph nodes.

Compared to other organs, bones have unique characteristics with respect to the TME [18]. In particular, adipocytes, one of the stromal cells, are rich in bone marrow, which has more chances to interact with tumor cells. Adipocytes in bone tissue release fatty acids used for energy production by metastatic cells, which lead to high expression of specific proteins, such as the fatty-acid-binding protein [129]. Alternatively, the metabolic phenotype of BM changes from glucose-avid to lipid-avid TME. Through this metabolic remodeling process, BM lesions tend to show high acetate uptake but low FDG uptake on dual-tracer PET/CT compared to metastatic lesions in other sites (Figure 4) [79].

**Figure 5:**
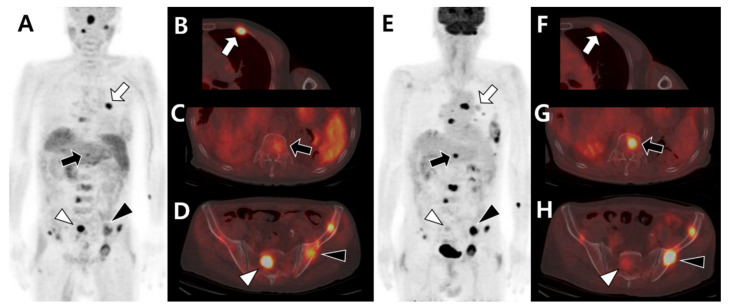
Different tracer avidities according to involved sites of bone metastases in the same patient with hepatocellular carcinoma before therapy. ^11^C-acetate positron emission tomography (PET)/computed tomography (CT) (**A**–**D**) shows intense acetate uptake in metastatic lesions of the left third rib (white arrow in **A**,**B**) and sacrum (white arrowhead in **A**,**D**) but mild acetate uptake in the T12 vertebra (black arrow in **A**,**C**) and left iliac bone (black arrowhead in **A**,**D**). ^18^F-fluorodeoxyglucose (^18^F-FDG) PET/CT (**E**–**H**) shows mild FDG uptake in the left third rib (white arrow in **E**,**F**) and sacrum (white arrowhead in **E**,**H**) but intense FDG uptake in the T12 vertebra (black arrow in **E**,**G**) and left iliac bone (black arrowhead in **E**,**H**).

Additionally, the image patterns using the same radiotracer can be different according to involved sites of BM or the presence of soft tissue involvement (Figure 5). ^18^F-FDG PET/CT showed higher FDG uptake in BM with bone destruction and soft tissue formation than in BM without soft tissue formation [77]. Metabolic assessments of BM using PET/CT have the potential value to predict or to determine the presence of BM-associated soft tissue formation, which can cause pain, spinal cord compression, and paralysis [130].

## 5. Conclusions

We reviewed well-used imaging tools for BM diagnosis and clinically available imaging modalities to visualize the bone TME. Additionally, we discussed how interactions between various factors affecting the TME were visualized using multimodal imaging techniques, focusing on BMs originating from thyroid and liver cancers.

The molecular imaging techniques to visualize the TME in BM are anticipated to expand their role to determine further therapeutic or management plans. Further research is necessary to assess multimodal imaging patterns to examine the TME with newly developed therapeutic targets (including cancer-associated fibroblasts [131] and neovasculature in tumor tissues [132]) in patients with BM from various types of cancers. In particular, multidisciplinary approaches are required for the optimization of management based on imaging-aided TME assessment for each metastatic lesion, even in the same patient. Through these efforts, multimodal imaging techniques are anticipated to prevent SREs and improve a patient’s quality of life and prognosis.

## Figures and Tables

**Table 1:** Major imaging patterns of bone metastasis according to the primary tumor.

Primary Tumor	Image Findings of Bone Metastasis			
	CT	MRI	Bone Scan	^18^F-FDG PET/CT
Thyroid	Osteoclastic activation [47] Osteolytic	Low SI in T1WI, high SI in T2WI [54]	Limited uptake in osteolytic predominant type [20,67,68]	Low FDG avidity in well-differentiated subtype [69] Hypermetabolic in poorly differentiated subtype [70,71]
Breast	Both osteoblast and osteoclast activation [59] Mixed	Low SI in T1WI, various SI in T2WI [38]	Increased uptake due to osteoblastic reaction [60,61]	Hypermetabolic in osteolytic predominant type [62,63,72,73]
Lung	Osteoclastic activation [50] Osteolytic	Low SI in T1WI, high SI in T2WI High SI on STIR image [52]	Increased uptake due to osteoblastic reaction [74] Limited uptake in osteolytic predominant type	Hypermetabolic in osteolytic predominant type [45,55,56,75]
Liver	Osteoclastic activation [48] Osteolytic	Low SI in T1WI, high SI in T2WI [53]	Limited uptake in osteolytic predominant type [76,77]	Low FDG avidity in well-differentiated type [58,78] Hypermetabolic in poorly differentiated type [77,79]
Kidney	Osteoclastic activation [49] Osteolytic	Low SI in T1WI, high SI in T2WI [51] High SI on STIR image [57]	Limited uptake in osteolytic predominant type [49,51] Increased uptake in compensatory osteoblastic activation [49]	Discernible FDG uptake in osteolytic type [80] Low FDG avidity in osteoblastic reaction [81]
Prostate	Osteoblastic activation [64] Osteosclerotic	Low SI in T1WI, various SI in T2WI [82]	Increased uptake due to osteoblastic reaction [66]	Low FDG avidity in osteosclerotic type [65,83,84]

CT: computed tomography; MRI: magnetic resonance imaging; ^18^F-FDG: ^18^F-fluorodeoxyglucose; PET: positron emission tomography; SI: signal intensity; T1WI: T1 weighted images; T2WI: T2 weighted images; STIR: short tau inversion recovery.

**Table 2:** Imaging modalities based on tumor microenvironment related factors.

TME-Related Factors	Imaging Mechanism	Imaging Modality	References
Tumor Metabolism			
Glucose	High expression of glucose transporters High glycolytic activity	^18^F-FDG PET/CT	[96,97]
Lipid	Increased lipid synthesis	^11^C/^18^F-acetate PET/CT	[98,99,100]
Nucleotide	Increased cellular proliferation and tyrosine kinases-1 activity	^18^F-fluorothymidine PET/CT	[101,102,103]
Cellular membrane	Increased choline transporters and choline kinase activity (cellular membrane turnover)	^11^C/^18^F-choline PET/CT	[104,105,106,107,108]
Amino acid	High expression of amino acid transporter and protein synthesis	^11^C-methionine PET/CT	[109,110]
Cellular density	Altered cellular density	MRI	[111,112]
Receptor expression			
Prostate-specific membrane antigen (PSMA)	High expression of PSMA in tumor	^68^Ga-PSMA PET/CT	[113,114,115,116]
Somatostatin receptor (SSTR)	High expression of SSTRs	^111^In-octreotide scintigraphy	[117,118]
Sodium iodide symporter (NIS)	High expression in differentiated thyroid cancer	^123^I/^131^I whole-body scintigraphy	[94,119,120]
Stromal cell activation			
Cancer-associated fibroblasts	High expression of fibroblast activation protein (FAP)	^68^Ga-FAPI PET/CT	[121,122,123]
Tumor-associated neovasculature	High expression of PSMA in the endothelium	^68^Ga-PSMA PET/CT	[116,124,125,126]
Bone marrow composition	Altered bone marrow composition	MRI	[127,128]

Abbreviations: TME: tumor microenvironment.

## Data Availability

Not applicable.
